# Effectiveness of an interactive telerehabilitation system with home-based exercise training in patients after total hip or knee replacement: study protocol for a multicenter, superiority, no-blinded randomized controlled trial

**DOI:** 10.1186/s13063-017-2173-3

**Published:** 2017-09-21

**Authors:** Sarah Eichler, Sophie Rabe, Annett Salzwedel, Steffen Müller, Josefine Stoll, Nina Tilgner, Michael John, Karl Wegscheider, Frank Mayer, Heinz Völler, on behalf of the ReMove-It study group

**Affiliations:** 1 Center of Rehabilitation Research, University of Potsdam, Am Neuen Palais 10, 14469 Potsdam, Germany; 2 University Outpatient Clinic, Center of Sports Medicine, University of Potsdam, Am Neuen Palais 10, 14469 Potsdam, Germany; 3 Fraunhofer Institute for Open Communication Systems, Kaiserin-Augusta-Allee 31, 10589 Berlin, Germany; 4 Department of Medical Biometry and Epidemiology, University Medical Center, Martinistrasse 52, 20246 Hamburg, Germany

**Keywords:** Telerehabilitation, Home-based, Total hip replacement, Total knee replacement, Exercise therapy, Aftercare

## Abstract

**Background:**

Total hip or knee replacement is one of the most frequently performed surgical procedures. Physical rehabilitation following total hip or knee replacement is an essential part of the therapy to improve functional outcomes and quality of life. After discharge from inpatient rehabilitation, a subsequent postoperative exercise therapy is needed to maintain functional mobility. Telerehabilitation may be a potential innovative treatment approach. We aim to investigate the superiority of an interactive telerehabilitation intervention for patients after total hip or knee replacement, in comparison to usual care, regarding physical performance, functional mobility, quality of life and pain.

**Methods/design:**

This is an open, randomized controlled, multicenter superiority study with two prospective arms. One hundred and ten eligible and consenting participants with total knee or hip replacement will be recruited at admission to subsequent inpatient rehabilitation. After comprehensive, 3-week, inpatient rehabilitation, the intervention group performs a 3-month, interactive, home-based exercise training with a telerehabilitation system. For this purpose, the physiotherapist creates an individual training plan out of 38 different strength and balance exercises which were implemented in the system. Data about the quality and frequency of training are transmitted to the physiotherapist for further adjustment. Communication between patient and physiotherapist is possible with the system. The control group receives voluntary, usual aftercare programs. Baseline assessments are investigated after discharge from rehabilitation; final assessments 3 months later. The primary outcome is the difference in improvement between intervention and control group in 6-minute walk distance after 3 months. Secondary outcomes include differences in the Timed Up and Go Test, the Five-Times-Sit-to-Stand Test, the Stair Ascend Test, the Short-Form 36, the Western Ontario and McMaster Universities Osteoarthritis Index, the International Physical Activity Questionnaire, and postural control as well as gait and kinematic parameters of the lower limbs. Baseline-adjusted analysis of covariance models will be used to test for group differences in the primary and secondary endpoints.

**Discussion:**

We expect the intervention group to benefit from the interactive, home-based exercise training in many respects represented by the study endpoints. If successful, this approach could be used to enhance the access to aftercare programs, especially in structurally weak areas.

**Trial registration:**

German Clinical Trials Register (DRKS), ID: DRKS00010009. Registered on 11 May 2016.

**Electronic supplementary material:**

The online version of this article (doi:10.1186/s13063-017-2173-3) contains supplementary material, which is available to authorized users.

## Background

In many industrialized countries, the number of hip and knee replacements is rising [1]. According to Organization for Economic Co-operation and Development (OECD) reports, 283 hip replacements and 190 knee replacements per 100,000 population were performed in Germany in 2013, which places Germany second and fourth worldwide, respectively [2]. Furthermore, due to an aging population and increasing obesity rates, a growing number of joint replacements can be expected [1].

After surgery, rehabilitation is an essential part of the therapeutic strategy and can improve function as well as activities of daily living and reduce pain [3]. In Germany, an orthopedic rehabilitation usually lasts 3 to 4 weeks and consists of multidisciplinary therapy elements [4].

The effectiveness of a subsequent rehabilitation for patients after hip or knee replacement is well proven [5–9], but its medium- and long-term sustainability to maintain the therapeutic success remains a major challenge. For this purpose, a subsequent exercise therapy after discharge from rehabilitation is needed [5, 10, 11], but recent data suggest [12] that only half of patients continue with recommended aftercare treatment options after the inpatient rehabilitation. Reasons for this could be the lack of reconciliation with job demands as well as long journeys to treatment-offering facilities. To improve sustainability of postoperative exercise therapy, there is a need for more flexible and individualized treatment options [12].

Telerehabilitation could have the potential to increase the access to therapy in structurally weak areas, where appropriate healthcare structures and offers are missing. Furthermore, telerehabilitation can be performed at any self-determined time and, therefore, could enhance the adherence and compliance, especially of employed patients.

There is an increasing evidence that orthopedic telerehabilitation has positive effects on various clinical conditions. First, investigations demonstrated non-inferiority for telerehabilitation interventions after knee replacement compared to face-to-face interventions [13–17]. However, the investigated telerehabilitation systems mainly differ in terms of implemented features. On the other hand, only insufficient data are currently available for the effectiveness of telerehabilitation for patients after hip replacement [18, 19]. Hence, the effectiveness of telerehabilitation systems needs to be investigated on each system itself. To our knowledge, no study in the German health system investigating the use of a telerehabilitation system in patients after total hip or knee replacement has been evaluated so far.

Therefore, the aim of this study is to investigate the effectiveness of an interactive telerehabilitation system with home-based exercise training considering the function of patients after total hip or knee replacement to ensure or increase the medium- and long-term sustainability of the inpatient rehabilitation success.

## Methods/design

### Study design

In this multicenter, no-blinded, parallel-group randomized controlled trial, patients after hip or knee replacement with a subsequent orthopedic rehabilitation are enrolled in the three participating inpatient rehabilitation centers in Germany. In this study, blinding was not possible due to the nature of the intervention. Assessments are carried out by research associates of University Outpatient Clinic of the University of Potsdam (study site), who are also responsible for giving out the telerehabilitation system to the patients of the intervention group.

**Fig. 1:**
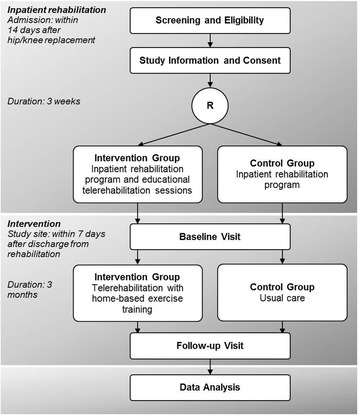
Study design

The patients are either randomized into the intervention or the control group, using a block randomization with a ratio of 1:1, stratified by the inpatient rehabilitation center. The random code was prepared by the statistical institute in advance and is centrally managed by the study site. The patients are investigated and assessed at the study site within 7 days after their inpatient rehabilitation (Fig. 1).

Patients are eligible for inclusion if a total hip or knee replacement after idiopathic, posttraumatic or congenital osteoarthritis was implanted, they are aged between 18 and 65 years and insured by the German Pension Insurance. Patients without expected functional safety in walking with full load at the end of the rehabilitation are excluded. For those patients, it is assumed that they are not able to perform exercises with adequate load and assessments at the study site. Insufficient skills of the German language in speech and writing also lead to exclusion. For the use of the telerehabilitation system at home, some additional criteria (e.g., High Definition Multimedia Interface (HDMI)-compatible screen, minimum 2.5 m space in front of the screen and Internet access) are required.

The study is conducted in accordance with the principles stated in the Declaration of Helsinki and Good Clinical Practice (International Conference of Harmonization). The study protocol was approved by the Ethics Committee of the University of Potsdam (No. 15/2016) and registered at the German Clinical Trials Register (DRKS00010009). The Standard Protocol Items: Recommendations for Interventional Trials (SPIRIT) checklist is available as an additional file (Additional file 1).

Data protection rules are closely observed and patient data are processed anonymously. All data are documented in paper and pencil form. Before the study, all assessments were determined in a standard operating procedure document including step-by-step instructions to promote data quality. For this purpose, every member of the staff was trained in advance. All data are entered electronically by a research assistant at the study site. The data entry file resembles the data collection paper form and contains programmed plausibility checks including referential data rules, valid value checks as well as range checks. Several research associates control the data independently.

### Inpatient orthopedic rehabilitation program

All patients undergo a subsequent 3- to 4-week, inpatient, orthopedic rehabilitation program in one of the three participating centers. The program starts within 14 days after surgery. The main components of the multidisciplinary therapy elements are exercise training and physiotherapy, training in activities of daily living, patient education, nutrition and psychological counselling as well as social support to increase patients’ ability to return to work [4]. Patients randomized into the intervention group have two additional 1-h educational sessions with a trained physiotherapist to become familiar with the telerehabilitation system. The latter took part in a structured “train-the-trainer” program in advance which included how to use the system, how to instruct the patients and how to supervise and monitor the intervention. During rehabilitation, potential confounding variables like 6-minute walk distance (6MWD) and range of motion of the joint are documented at admission and at discharge of rehabilitation.

### Intervention

The intervention consists of a 3-month, interactive telerehabilitation with home-based exercise training and is based on the system MyRehab®. It starts within 7 days after discharge from inpatient rehabilitation.

The individually tailored exercise training regarding the choice of exercises (38 different strength and balance exercises) and training modalities (number and sets of repetitions as well as duration of resting time) is created and monitored by the responsible physiotherapist in the rehabilitation center. The patient is instructed to exercise 3 to 4 times a week. The physiotherapist and the patient are always able to communicate via text or voice messages and a video-conference is scheduled on a weekly basis.

The exercises are shown on a screen and the patient is instructed to perform the exercises simultaneously with the system. While exercising, the patient is recorded by a video camera. By tracking the movement pattern, the patient receives an automatic real-time motion feedback in the form of green and red coloration of the single body segments for correct and incorrect movements, respectively. After each exercise, the patient gets a grade evaluating the quality of each exercise as well as the whole training, which can also be seen and interpreted by the physiotherapist in the rehabilitation center for further adjustment of the exercise training.

Patients in the intervention group are allowed to use additional, voluntary, usual aftercare programs, such as exercise-based group training, individualized exercise training or physiotherapy, or to exercise on their own. All performed training options including frequency and duration are recorded in a personal training diary.

### Control condition

Patients randomized into the control group receive voluntary usual care after their inpatient rehabilitation which includes the usual aftercare programs, such as exercise-based group training, individualized exercise training or physiotherapy, or to exercise on their own. Everything is also recorded in a personal training diary. To promote patient retention, especially in the control group, patients are offered an evaluation of their investigations at the study site. Additionally, all patients receive a phone call 1 week before follow-up.

### Assessments

**Fig. 2:**
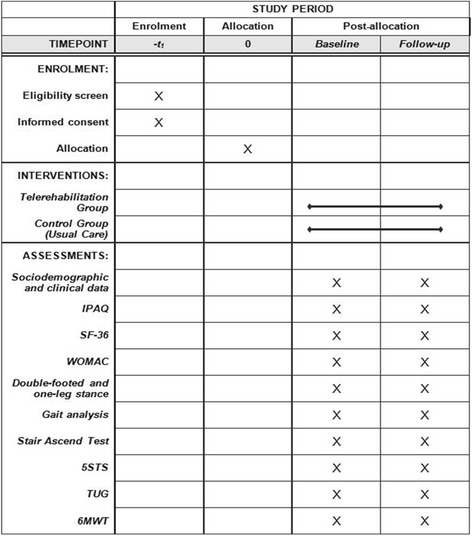
Standard Protocol Items: Recommendations for Interventional Trials (SPIRIT) Figure. *IPAQ* International Physical Activity Questionnaire, *SF-36* Short Form 36, *WOMAC* Western Ontario and McMaster Universities Arthritis Index, *5STS* Five-Times-Sit-To-Stand Test, *TUG* Timed Up and Go Test, *6MWT* 6-minute walk test

During the baseline visit, sociodemographic (e.g., age, sex, weight, gender, educational level and work ability) and clinical data (e.g., risk factors, comorbidities, pain medication and data of surgery) are documented. Furthermore, the following assessments are performed at baseline and at follow-up 3 months later: functional assessments such as the 6-minute walk test (6MWT) [20] for physical capacity, the Timed Up and Go Test (TUG) [21], the Stair Ascend Test [22], the Five-Times-Sit-to-Stand Test [23] for balance, strength and mobility, the questionnaires Short Form 36 (SF-36) [24] for health-related quality of life, International Physical Activity Questionnaire (IPAQ) [25] for the level of physical activity and the Western Ontario and McMaster Universities Arthritis Index (WOMAC) [26] for pain and stiffness. Measures of function include the assessment of the postural control during double-footed and one-leg stance [27] for left and right leg (barefoot, hands to the hip, view straight forward) for 30 s on a force plate (Amti OR6-6, Advanced Mechanical Technology, Inc. Watertown, MA, USA) as well as gait analysis during over-ground walking (10 trials) with self-selected velocity using a 3D-motion analysis system (12 cameras, Vicon MX; 200 Hz; Vicon Ltd., Oxford, UK) and a kinematic Plug-in Gait lower body model (Vicon Nexus) [28] (see Fig. 2 for the Standard Protocol Items: Recommendations for Interventional Trials (SPIRIT) Figure that the trial follows). Additionally, adverse events (e.g., wound infection, joint luxation) will be documented and evaluated. Further adverse events are not expected due to the constitution of the patients after the inpatient rehabilitation.

### Primary and secondary outcomes

The primary outcome is the difference in enhancement of the 6MWD between intervention and control group in meters after 3 months. The 6MWT is an objective assessment [20] that has been found to detect functional improvement after total hip and knee replacement [29–32].

To provide a comprehensive comparison between the two groups, a battery of questionnaires, functional and measures of function will also be investigated:

### Differences in the improvement of the:

- Return to work status
- IPAQ score (points)
- SF-36 Physical and Mental Component Summaries (points)
- WOMAC score (points)
- Postural control (double-footed and one-leg stance): the overall displacement of the center of pressure (mm) for the 30 s (first 10 s) time interval
- Gait analysis: gait velocity (m/s); step length/width (cm); maximal, minimal and mean ankle, knee, hip joint angles (°)
- Stair Ascend Test (s)
- Five-Times-Chair-Rise Test (s)
- Timed Up and Go Test (s), and
- Training adherence after the intervention time of 3 months

### Statistics and power calculation

Data will be evaluated descriptively (mean, standard deviation, 95% confidence interval and median for metric variables, frequency and percentiles for categorical variables) for all variables as available case analysis. Inferential statistics will be performed using an intention-to-treat approach including all randomized patients and with multiply imputed data sets for evaluation of the effectiveness of the intervention, as well as a per-protocol approach in patients with complete data sets and without protocol violations for evaluation of the efficacy of the intervention. Baseline-adjusted analysis of covariance (ANCOVA) models (intention-to-treat) will be used to test the primary endpoint for group differences at a level of 5% (two-sided). All continuous secondary endpoints will be analog-tested without adjustment for multiplicity. The rates of return to work status will be compared with the Likelihood-Ratio-Chi^2^-Test. Model assumptions will be checked by testing site × group interactions as extensions of the analysis of covariance (ANCOVA) models.

According to Boardman et al. [30], an improvement of 65–70 m in 6MWD is to be expected by knee or hip replacement followed by standard orthopedic rehabilitation. The study was powered to be able to demonstrate a further improvement of 65 m by telerehabilitation as compared to an improvement of not more than 13 m (20%) in the control group. Assuming an intraclass correlation of 0.3 for the primary outcome and a standard deviation of 84 m [30, 33], 42 patients per group will be needed to reach a power of 80% for the primary analysis. Presuming a 20–25% loss to follow-up, 55 patients per group need to be included in the study to reach the same power in the per-protocol analysis, resulting in a recruitment and randomization of 110 patients in total.

## Discussion

The medium- and long-term sustainability of maintaining the therapeutic success of rehabilitation programs remains a major challenge. Treatment options after rehabilitation are often far away and difficult to access. Therefore, telerehabilitation could have the potential to increase the access to therapy in structurally weak areas, where appropriate healthcare structures and offers are missing, as it promises to increase patient access, improve quality and reduce costs in healthcare [13]. For patients, especially after orthopedic surgery, telerehabilitation seems to be a promising offer for the recovery of motor function [17, 34] which might be useful for longer distances to work and for coping with challenges of everyday life. Therefore, we concentrate on functional outcomes, such as the 6MWT, as the primary outcome to prove the effectiveness of our telerehabilitation system. The 6MWT is a strong predictor for mobility and functional recovery after hip or knee replacement [30, 31]. To achieve a complex representation of both functional and emotional status, which need to be considered in a rehabilitation process, further investigation of the secondary endpoints to show differences in mobility, strength, postural control as well as health-related quality of life and pain seems appropriate.

It is well known that the compliance of patients performing home-based exercises needs to be improved and the flexible use of telerehabilitation could increase the adherence [35]. The reasons for the non-compliance of the patients are suggested to be the absence of positive feedback as well as a degree of experienced helplessness [36]. Solutions for this problem could be setting goals, being monitored and receiving feedback [37] by using telerehabilitation systems. In our study, we expect a strong adherence in the intervention group due to the close monitoring of the supervising physiotherapist in the rehabilitation center, who is familiar with the patients because of their previous inpatient stay. With scheduled, weekly video-conferences and the steady possibility to communicate via text or voice messages, the patients might greatly benefit regarding the motivational component of being physically active. We are able to examine the adherence of the patients with the training diary, which every patient in both groups has to fill out weekly. It is also conceivable that the improvements in the control group result from more elaborate program and treatment frequency of the voluntary usual aftercare.

Due to the fact that blinding of the research associates is not possible, we cannot rule out that the patients could possibly be influenced during the measurements. Furthermore, we need to exclude patients without expected functional safety in walking with full load at the end of the rehabilitation, which might be a selection bias. This patient selection compromises the generalizability of the results, but presumably does not bias the comparison between random groups.

In conclusion, we expect the intervention group to benefit from the interactive, home-based, exercise training concerning the outcome. If successful, this approach could be used to enhance the access to aftercare programs, especially in structurally weak areas.

## Trial status

Recruitment has begun in August 2016. Up to now (05/2017), 87 patients are included.

## Individual gratitude

**Table:** 

Study site, technology partner and rehabilitation centers		
Project management Center of Rehabilitation Research, University of Potsdam		
Völler Heinz, Prof. Dr.	Salzwedel Annett, Dr.	Eichler Sarah
Rabe Sophie	Freitag Nils	Hadzic Miralem
Discher Robert		
University Outpatient Clinic Potsdam, University of Potsdam		
Mayer Frank, Prof. Dr	Müller Steffen, Dr.	Cassel Michael, Dr.
Brecht Pia	Herber Mike	Krause Petra
Stoll Josefine	Wochatz Monique	Tilgner Nina
Koalick Karolin	Schubert Daniela	Henschke Jakob
Fraunhofer Institute for Open Communication Systems		
John Michael, Dr.	Klose Stefan, Dr.	Häusler Benny
Schröder Cornelia	Giertz Christian	
MEDIAN Klinik Hoppegarten		
Krause Matthias, Dr.	Thomas Frank, Dr.	Messer Jana, Dr.
Haupt Thomas	Scholz Ina	
Brandenburgklinik Bernau		
Reich Horst, Dr.	Limburg Hardy, Dr.	Haendel Heiko, Dr.
Stuckert Olga	Farschatov Natalia	Lade Juri
Nachef Raymond	Weisheit Mandy	Kascha Thomas
Bork Heike	Unger Martina	Zeiske Chris
Vorwerk Christel	Benoit Kathrin	Kandula Kathrin
Rehazentrum Lübben		
Hentschke Mike-Percy, Dr.	Öksus Göhnen	Tsadrafilis Emmanuel
Liebach Jana	Wolschke Mirko	Smurawski Andreas
Department of Medical Biometry and Epidemiology, University Medical Center Hamburg		
Wegscheider Karl, Prof. Dr.	Balzer Klaus	

## Additional file

Additional file 1: SPIRIT Checklist. Overview of important items of a clinical trial and their placement in the manuscript. (PDF 169 kb)

## Abbreviations

5STS: Five-Times-Sit-To-Stand Test
6MWD: 6-minute walk distance
6MWT: 6-minute walk test
ANCOVA: Analysis of covariance
HDMI: High Definition Multimedia Interface
IPAQ: International Physical Activity Questionnaire
OECD: Organization for Economic Co-operation and Development
SF-36: Short Form 36
SPIRIT: Standard Protocol Items, Recommendations for Interventional Trials
TUG: Timed Up and Go Test
WOMAC: Western Ontario and McMaster Universities Arthritis Index
